# The Effect of Pre-Drying Treatment and Drying Conditions on Quality and Energy Consumption of Hot Air-Dried Celeriac Slices: Optimisation

**DOI:** 10.3390/foods10081758

**Published:** 2021-07-29

**Authors:** Tina Nurkhoeriyati, Boris Kulig, Barbara Sturm, Oliver Hensel

**Affiliations:** 1 Department of Agricultural Engineering, Faculty of Organic Agricultural Sciences, University of Kassel, 37213 Witzenhausen, Germany; bkulig@uni-kassel.de (B.K.); agrartechnik@uni-kassel.de (O.H.); 2 Study Program of Food Technology, Faculty of Life Sciences, International University Liaison Indonesia, Tangerang 15345, Indonesia; 3 Leibniz Institute for Agricultural Engineering and Bioeconomy (ATB), 14469 Potsdam, Germany; BSturm@atb-potsdam.de; 4 Albrecht Daniel Thaer Institute for Agricultural and Horticultural Sciences, Humboldt Universitӓt zu Berlin, 10115 Berlin, Germany

**Keywords:** antioxidant activity, browning index, celeriac, energy demand, hot air drying, optimisation, rehydration ratio, total colour difference, total phenolic compound

## Abstract

Celeriac is a good source of fibre, trace minerals, and phenolic compounds; it has a pleasant aroma but is a perishable material, prone to discolouration. This research investigated the optimisation of the quality and energy demand in hot-air dried celeriac slices. The experiment utilised the I-optimal design of response surface methodology with 30 experiment runs. Pre-drying treatments (blanching at 85 °C, three minutes; dipping in 1% citric acid solution, three minutes; no pre-drying treatment), drying temperatures (50, 60, and 70 °C), air velocities (1.5, 2.2, and 2.9 m/s), and thickness (three-, five, and seven-mm) were applied. The drying conditions affected drying time significantly (*p* < 0.0001). The model by Midilli and others and the logarithmic model fitted best with celeriac slices drying kinetics. Blanched samples had a higher ΔE*_ab_ (total colour difference) and *BI* (browning index) but lower *WI* (whiteness index) than samples with other pre-drying treatments. The rehydration ratio decreased with the increase of sample thickness and blanching (*p* < 0.0001). A quadratic model described the specific energy consumption (*E_s_*) best. The dried samples compared with fresh samples had increased antioxidant activity but decreased total phenolic compound value. The optimisation solution chosen was 58 °C drying temperature, 2.9 m/s air velocity, and 4.6 mm sample thickness with acid pre-drying treatment.

## 1. Introduction

Food manufacturers use drying as a primary technique in the preservation of perishable products. Drying allows the products to be available regardless of the season [1]. In addition, this process has the main objective of lowering water activity below 0.5 to inhibit microorganisms’ growth [2]. Hot-air drying is the most widely used artificial drying technology [3].

Drying offers many benefits for food products, such as reduced weight and volume, to lower transportation costs and extend product shelf life without refrigeration. Furthermore, it produces intermediate or convenience food products. Drying consists of complex mechanisms, such as physical, chemical, and biochemical reactions, making it an unsteady, extremely non-linear, and dynamic, thermal process [4]. However, drying may also lead to undesirable quality changes [2], such as altering food quality parameters, such as colour [5,6,7,8,9,10], shrinkage [6,11], rehydration ratio [12], antioxidant activities [7,12,13,14], and total phenolic compound [5,7], to name but a few.

Celery and celeriac (*A. graveolens*) are aromatic vegetables produced worldwide with essential pharmacological attributes [15]. Celeriac (*A. graveolens var. rapaceum*), also known as root celery or turnip-rooted celery, is popular in Europe. The European Union (EU) produced 490,080 t celeriac in 2019 [16].

Celeriac is a good source of fibre, trace mineral, and phenolic compounds; it has a pleasant characteristic aroma but is prone to discolouration [17,18]. The latter case may influence the sensory quality and reduce consumer acceptance. Furthermore, celeriac is a wet perishable material due to its high moisture level of 88% (wet basis) [19]. The consequence of this high moisture content is that the product is vulnerable to deterioration. Therefore, drying enables celeriac to be more easily handled and transported, and for it to have an extended shelf-life. Moreover, after a further process, such as milling, the resulting dried celeriac result in flour for ingredients in various food products [20], and functional food development, e.g., high fibre products [21].

Researchers have carried out several studies related to celeriac drying with various quality attributes observed. The attributes studied included mass transfer coefficient and effective moisture diffusivity [22,23,24], heat transfer coefficient [25], drying period [24,26], energy consumption [26], dry matter, moisture content, and diffusion coefficient [27], drying rate [24,27,28], moisture content and moisture ratio [24], colour [26,28], rehydration and shrinkage [28], and natural antioxidants changes [29].

The studies paid particular attention to the evaluation of thermal properties. However, research regarding the optimisation of quality and energy demand in celeriac hot-air drying is not yet available. Meanwhile, it is essential to get insights into antioxidant changes during vegetable processing, such as drying, because vegetables are essential antioxidants (e.g., vitamin C, E, phenolic compounds) sources [29]. Furthermore, drying efficiency involves energy consumption, drying rate, and drying time to determine drying performance [30]. Hence, investigating the effect of the factors (drying temperature, air velocity, sample thickness, pre-drying treatment) on celeriac slices’ quality and energy consumption during hot-air drying is essential.

## 2. Materials and Methods

### 2.1. Plant Material

Organic celeriac (*Apium graveolens* var. *Rapaceum*) for this research was obtained from a local farmer in Göttingen, Germany. The roots were harvested on 15th November 2020, stored at a temperature of 1 °C, and utilised in the experiment in January 2021.

### 2.2. Design of Experiment

This study investigated the effect of drying conditions (temperature, air velocity, sample thickness) and pre-drying treatments on drying time, quality (chromaticity, rehydration ratio, retention of antioxidant activity, retention of total phenolic compounds), and specific energy consumption. The experiment used the I-optimal design of response surface methodology (Design-Expert^®^ version 12, Stat-Ease, Inc., Minneapolis, MN, USA) with 30 experiment runs (18 required model points, two additional model points, five lack-of-fit points, and five replicate points). I-optimal designs focus on designs that minimise the average prediction variance over the experimental space [31]. Equation (1) describes the common second-order model used in response surface methodology (RSM). Table 1 summarises the factors in this study.

(1) $$y=\beta_{0}+\sum_{i=1}^{k}\beta_{i}x_{i}+\sum_{i}\sum_{j}\beta_{ij}x_{i}x_{j}+\sum_{i=1}^{k}\beta_{ii}x_{i}^{2}+\varepsilon ,$$

where *y* is the response function; *β_i_*, *β_ii_*, and *β_ij_* are the coefficients of the linear, quadratic, and interaction terms, respectively; *x_i_* and *x_j_* are the factors or independent variables, *k* is the number of variables, and *ε* is error term [32].

This study fitted the experimental data to the statistical model (linear, two factors interaction, quadratic or cubic model). Models were compared based on their coefficient of determination (R^2^), adjusted coefficient of determination (R^2^-adj), and predicted coefficient of determination (R^2^-pred). After selecting the adequate model, the investigators used the variance (ANOVA) analysis to investigate the statistical significance of the regression coefficients at a 95% confidence level. All non-significant (*p* > 0.05) terms were eliminated from the model, not counting those required to support hierarchy. A desirable model is a model which is significant (*p* < 0.05) with no significant Lack of Fit (*p* > 0.05), post-ANOVA statistics with R^2^-adj − R^2^-pred < 0.2, and an adequate precision > 4 [33].

### 2.3. Sample Preparation and Pre-Drying Treatment

The celeriac was washed, peeled, and sliced using a bench slicer (GRAEF GmbH, Berlin, Germany) to obtain the desired thickness level (three, five, or seven millimetres). Slices produced were cut to get pieces of 30 mm in diameter. Each drying run utilised a total of 12 celeriac slices (four slices for moisture content and colour analysis, four slices for rehydration ratio, and four slices for chemical analysis). Four pieces of fresh samples were measured for moisture content and chromaticity, and four slices for chemical analysis. For pre-drying treated samples, the moisture content was also recorded. The initial total weight of celeriac dried for three-, five-, or seven-mm initial thickness was 29.10 ± 1.12 g, 45.62 ± 2.25 g, and 62.50 ± 1.02 g, respectively.

The celeriac slices were put on the perforated shelves of the dryer. The dryer has 420 mm × 440 mm × 470 mm (HT Mini, Innotech Ingenieursgesellschaft mbH, Altdorf, Germany) dimensions and was turned 30 min before each drying experiment to reach the steady-state condition. Pre-drying treatments applied for celeriac slices were blanching and acid dipping. The blanching was done at 85 °C in a water bath (Memmert GmbH + Co. KG, Schwabach, Germany) for three minutes, which was immediately stopped with cold distilled water, and putting them on the blotting paper to remove excess water [28]. Acid dipping was done by immersing the samples in 1% citric acid solution at room temperature for three minutes and putting them on the blotting paper to remove excess water. Citric acid is the most widely used anti-browning acid because of its availability, price, and neutral impact on product sensory attributes [34]. Moreover, citric acid is considered an organic acid that allows pre-treatment organic food of plant origin [35]. Therefore, samples without pre-drying treatment were also prepared.

Drying experiments were performed at three levels of inlet temperatures of 50, 60, and 70 °C, which are commonly used in food drying because they are sufficient to promote water evaporation from the samples and, at the same time, preserve their volatile compound [36,37]. The levels of inlet air velocity of 1.5, 2.2, and 2.9 m/s were applied. A thickness of three-, five, and seven-mm was used. This range covered most thickness use in celeriac drying [23,24,25,26,27,28,38]. The final moisture content target was 10 g water/100 g sample (wet basis) or 0.11 g water/g dry solid (dry basis), which is sufficient to extend the shelf life of dried vegetables [39]. Samples taken for chemical analysis were vacuum-packed and stored at −28 °C for further analysis.

### 2.4. Drying Time (D_t_), Drying Rate (DR), Moisture Content (MC), and Moisture Ratio (MR)

Celeriac samples were weighed every 20 min during the initial two hours of the drying process, and hourly after that, to record the moisture weight reduction. The moisture content of the dried celeriac at the end of drying was determined by drying the samples at 105 °C for 24 h in the oven (ULM 400, Memmert GmbH + Co. KG, Schwabach, Germany) [40] to obtain the dry matter percentage of the dried celeriac. Drying time (*D_t_*) is the time needed to dry a fresh sample until it reaches a final moisture content of 0.11 g water/g dry solid.

Moisture content (*MC*) was determined as calculated using Equation (2) and expressed as g water/g dry solid (g_w_/g_ds_).

(2) $$MC=\frac{W_{0}-W_{t}}{W_{t}\;}.$$

*W*_0_ and *W_t_* are the weight of the initial sample and the dried sample. A dimensionless moisture ratio (*MR*) avoids ambiguity because of the initial *MC* difference among samples [28]. *MR* and the drying rate (DR) of celeriac drying were determined using Equations (3)–(5), as follows:

(3) $$MR=\frac{MC_{t}-MC_{e}}{MC_{0}-MC_{e}}.$$

*MC_e_* is negligible compared to *MC_t_*, *MC*_0_ during long-term drying [41]; only the first term is considered to be significant [42]. Thus, the formula becomes:

(4) $$MR=\frac{MC_{t}}{MC_{0}},$$

(5) $$DR\;\left(g_{w}/g_{ds}.min\right)=\frac{MC_{t}-MC_{t+\Delta t}}{\Delta t}.$$

*MC_t_*, *MC*_0_, *MC_e_*_,_ *MC_t+_*_Δ_*_t_* are moisture content of the sample at any given time, at the initial, at the equilibrium, and at a specific time *t+*∆*t* on a dry basis, respectively [24].

### 2.5. Drying Kinetics Model Fitting

The drying kinetics curves of each experiment run were constructed by plotting drying time against moisture ratio. The drying experiments were fitted to 11 mathematical models commonly used for thin-layer drying [41]. Those mathematical models are semi-theoretical (Lewis, Page, Modified Page, Modified Page equation-II, Henderson and Pabis, Logarithmic, Approximation of diffusion, Midilli and others, Verma and others, Modified Henderson and Pabis) and empirical model (Wang and Singh) [41]. The use of these 11 mathematical models compared to fewer numbers had the advantage of having a greater chance of finding mathematical models applicable in describing the behaviour of celeriac during the drying process.

JMP^®^ software version 15.2.0 (SAS Institute, Inc., Cary, NC, USA, 1989–2019) column formula function was assigned to analyse the non-linear regression and determine the model coefficient. We selected the best-fitted model based on the lowest Sum squared of Error (SSE) at the 0.05 level of significance [43]. SSE was calculated using Equation (6). Drying kinetics graph generation in this paper utilised Matplotlib: A 2D graphics environment [44].

(6) $$SSE=\sum_{i=1}^{n}{\left(MR_{t}-{\hat{MR}}_{i}\right)}^{2},$$

where ${\hat{MR}}_{i}$ is the predicted moisture ratio.

### 2.6. Chromaticity

Celeriac chromaticity was measured every 20 min for the first two hours during drying, and then hourly, until the end of the process. Chromaticity changes measurement utilised the same samples as for the moisture content measurement. The chromaticity indices (L*, a*, and b* values) of each dried celeriac slice were measured in triplicate using a Minolta chromameter (model CR-400, Minolta Camera Co., Ltd., Osaka, Japan) [45]. CIE L*a*b* colour space difference represents colour difference perceived by humans [46].

Several chromaticity indices were determined. Those indices under consideration were whiteness index (*WI*) [47], browning index (*BI*) [48], and total colour difference (ΔE*_ab_) [49], and they were calculated using Equations (7)–(10) below:

(7) $$WI=100-\sqrt{{\left(100-L^{*}\right)}^{2}+a^{*}{}^{2}+b^{*}{}^{2}},$$

(8) $$BI=\frac{\left[100\;\left(x-0.31\right)\right]}{0.172},$$

where:

(9) $$\frac{x=\left(a^{*}{}_{t}+1.75L^{*}{}_{t}\right)}{5.645L^{*}{}_{t}+a^{*}{}_{t}-3.012b^{*}{}_{t}},$$

(10) $$\Delta E^{*}{}_{ab}=\sqrt{{\left(L^{*}{}_{t}-L^{*}{}_{0}\right)}^{2}+{\left(a^{*}{}_{t}-a^{*}{}_{0}\right)}^{2}+{\left(b^{*}{}_{t}-b^{*}{}_{0}\right)}^{2}}.$$

ΔE*_ab_ is the total colour difference (dimensionless), *BI* is the browning index (dimensionless), *WI* is whiteness index (dimensionless), L* is lightness coordinate, a* is red/green coordinate, b* is yellow/blue coordinate, subscript 0 indicates the initial value of the parameter, and subscript *t* indicates the value of the parameter at time t. Drying kinetics graph generation in this paper utilised Matplotlib: A 2D graphics environment [44].

### 2.7. Rehydration Ratio (RR)

The sample was placed in the beaker glass, and cold distilled water was added with a ratio of 1:40, and then it was covered with a watch glass. Then, the water was brought to a boil on the electric heater and boiled gently for five minutes. Then, the beaker was removed from the heater, and the water was drained. The rehydrated sample was kept over a sheet of blotting paper, to remove excess moisture adhering to surfaces, and quickly weighed [50]. The *RR* (dimensionless) was expressed as following Equation (11):

(11) $$RR=\frac{W_{r}}{W_{0}}.$$

*W_r_* and *W*_0_ are the weight (g) of drained samples after rehydration and fresh samples, respectively.

### 2.8. Antioxidant Activity (AA)

The fresh and the dried celeriac were subjected to DPPH free radical spectrophotometric assay for their antioxidant activity assessment. The analysis followed the procedure introduced by Brand-Williams et al. [51]. The extraction followed the process as described by Karabacak et al. [38], with modification. The dried samples were freeze-dried (Freeze-dryer Alpha 1-2 LDplus, Martin Christ Gefriertrocknungsanlagen GmbH, Osterode am Harz, Germany) for 48 h (condenser temperature: −30 °C; chamber pressure: 0.37 mbar). The extract of dried celeriac was obtained by mixing homogenised freeze-dried samples with HCl_conc_/methanol/water (1:80:10, *v*/*v*) mixture with 1:10 ratio and shaken for two hours at room temperature at 150 rpm (Kottermann, GmbH, Uetze, Germany). Then, the mixture was centrifuged at 6000 rpm for five minutes at room temperature (ROTILABO^®^, Carl Roth, Karlsruhe, Germany). The analysis used 2,2-diphenyl-1-picrylhydrazyl (DPPH) (Merck, Taufkirchen, Germany) (molecular mass of 394.32 g/mol) reagent as a radical solution obtained by dissolving 1.182 mg DPPH in methanol (ROTIPURAN^®^ ≥ 99.9% p.a. ACS, ISO, Carl Roth, Karlsruhe, Germany) to obtain 50 mL solution with 6 × 10^−5^ M concentration. Then, 3.90 mL of DPPH radical solution was mixed with 0.1 mL of methanolic solutions of samples and shaken using vortex for 10 s. The mixture was then incubated for 30 min at room temperature. After incubation, the absorbance decrease of the mixture was measured at 515 nm (Spectronic GENESYS 10 S UV—Vis Scanning Thermo Scientific™, Thermo Fisher Scientific, Inc., Waltham, MA, USA). Trolox was used as the positive control. Calibration was done by measuring the methanolic solution of (S)-6-Methoxy-2,5,7,8-tetramethylchromane-2-carboxylic acid (Trolox) (Merck, Taufkirchen, Germany) with the concentrations of 0–1200 µM. The antioxidant activity result was expressed as µmol Trolox equivalent/g dry solid sample (µmol TE/g_ds_). Retention of *AA* (%*_AA_*) was described as in Equation (12).

(12) $${\%}_{AA}\;\left(\%\right)=\frac{AA_{t}}{AA_{0}}\times 100\%.$$

### 2.9. Total Phenolic Compound (TPC)

The phenolic compound is one of the nutritional quality benchmarks during drying [52]. Samples for the total phenolic compound were obtained from methanolic extract as made previously. The procedure followed Odeh et al. [53], with minor modification. Celeriac extract or gallic acid standard (100 µL) were added with 1.8 mL of ten times distilled water-diluted Folin–Ciocalteu reagent (Th. Geyer GmbH & Co., KG, Hamburg, Germany). The mixture was rested for five minutes at room temperature and then mixed with 1.2 mL of sodium bicarbonate (7.5%, *w*/*v*) (≥99.5% p.a. ACS, ISO, Carl Roth, Karlsruhe, Germany). Afterwards, the samples were stood for 60 min at room temperature and dark condition. Finally, the samples’ absorbance was measured at 765 nm. Calibration was done by measuring an aqueous solution of gallic acid (Merck, Taufkirchen, Germany) with 0–500 mg/L concentrations. The total phenolic compound result was expressed as mg gallic acid equivalents/g dry solid (mg GAE/g_ds_). Retention of *TPC* (%*_TPC_*) was computed as in Equation (13).

(13) $${\%}_{TPC}\;\left(\%\right)=\frac{TPC_{t}}{TPC_{0}}\times 100\%.$$

### 2.10. Specific Energy Consumption (E_s_)

Total energy consumption (*E_s_*) was computed according to Equation (14) [54] as follows:

(14) $$E_{t}=A\times \upsilon \times \rho_{a}\times c_{a}\times \Delta T\times D_{t}.$$

*E_t_* is the total needed energy for drying; *A* is tray area; *ν* is air velocity; *ρa* is air density; *c_a_* is the specific heat; Δ*T* is temperature differences; *D_t_* is whole drying time. While specific energy consumption was expressed as in Equation (15) below:

(15) $$E_{s}=\frac{E_{t}}{W_{0}}.$$

*E_s_* is specific energy consumption (kJ/kg), and *W*_0_ is the initial weight (kg).

### 2.11. Optimisation

This study applied numerical optimisation, which found a point with a maximum desirability function (optimum set condition). In addition, propagation of error (POE) was considered. POE plots showing how that error is transmitted to the response [55] to seek a robust formula for slight variations in the measured amounts by minimising transmitted variation [56].

Desirability, an objective function, optimises multiple response measures simultaneously [57]. This function can be optimised by univariate techniques to obtain an optimal solution and avoid investigators making trade-offs because of conflicting multiple response measures [58].

Derringer and Suich [59] modified the previously proposed desirability function approach by Harrington [60]. They outlined the desirability function by transforming each estimated response variable y_i_ to a desirability value *d_i_*, 0 (not acceptable) ≤ *d_i_* ≤ 1 (complete satisfaction). The investigator can assign *d_i_* to minimise, maximise, or aim to a target value [58]. Individual desirability is then combined to result in the overall desirability as computed by geometric mean, as in Equation (16) below [59]:

(16) $$D={\left(d_{1}\times d_{2}\times \ldots \times d_{k}\right)}^{1/k}.$$

*D* is overall desirability, *d* is individual desirability, and *k* is the response variable.

## 3. Results and Discussion

### 3.1. Effect of Drying Factors on Hot Air-Dried Celeriac Slices Drying Kinetic

**Figure 1:**
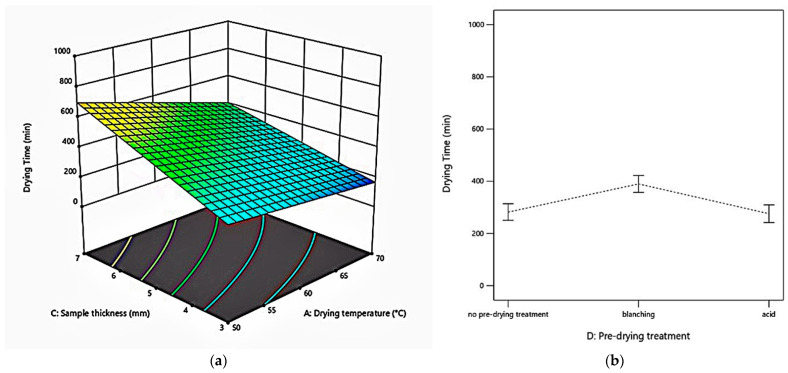
Drying time as a function of factors: (**a**) Drying time of celeriac slices at different drying temperatures and sample thicknesses, air velocity of 1.5 m/s, and blanching pre-drying treatment.; (**b**) drying time of celeriac slices at various pre-drying treatments, sample thickness of 5 mm, air velocity of 1.5 m/s, and drying temperature of 60 °C.

The moisture content of samples without pre-drying treatment (N), blanched (B), or acid dipped (A) is 6.96 ± 0.58 g_w_/g_ds_, 7.51 ± 0.28 g_w_/g_ds_, 8.77 ± 0.79 g_w_/g_ds_, respectively. The average final moisture content at all experiment runs was 0.11 ± 0.01 g_w_/g_ds_. Drying time ranges from 100 min (60 °C, 2.9 m/s, 3 mm, acid) to 810 min (50 °C, 1.5 m/s, 7 mm, blanching). Figure 1 shows drying time as a function of factors.

As expected, the drying time was directly proportional to the thickness of the sample (*p* < 0.0001), but, with the increase of the temperature (*p* < 0.0001) and drying air velocity (*p* < 0.001), it declined. This finding is aligned with the results of other reports on celery root [24,26], carrot [61,62], potato [63], quinces [64], and pumpkin [65].

**Figure 2:**
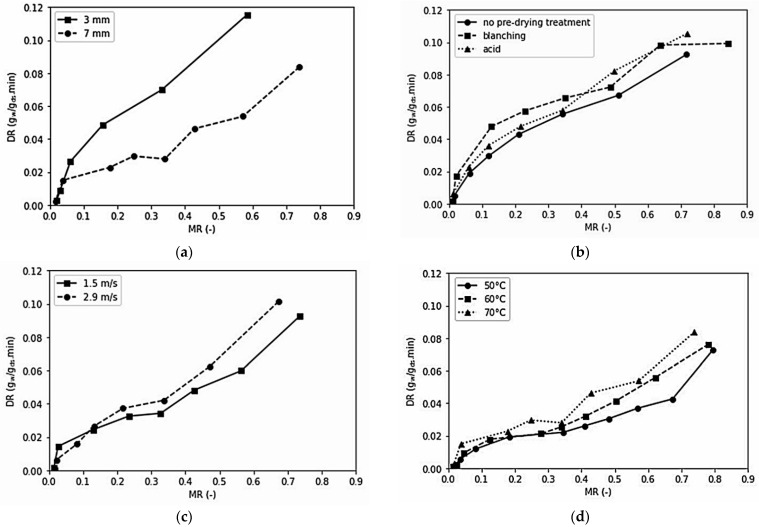
Drying rate as a function of moisture ratio at (**a**) 70 °C drying temperature, 2.2 m/s air velocity, no pre-drying treatment; (**b**) 50 °C drying temperature, 1.5 m/s air velocity, 3 mm sample thickness; (**c**) 60 °C drying temperature, 5 mm sample thickness, no pre-drying treatment; (**d**) 2.2 m/s air velocity, 7 mm sample thickness, no pre-drying treatment.

Thinner slices have less distance for the moisture to travel from the centre to the surface and have a larger surface and volume ratio, requiring a shorter time to dry [66]. As a result, the drying rate of thinner slices was higher than that of the thicker ones, as shown by Figure 2a. In addition, higher temperature and air velocity led to increased moisture removal rate, as shown in Figure 2c,d, so dried samples have a shorter time to attain final moisture content [67].

Pre-drying treatments affected the drying time (*p* < 0.0001). Blanching increased the drying time. The blanched samples had higher initial moisture content than other samples, requiring a longer drying time to achieve a certain final moisture content, even though the drying rate was relatively higher than other treatments (Figure 2b). Ndisya et al. [68] also found that blanching pre-treated cocoyam had a longer drying time than non-treated samples. They hypothesised that the application of water soaking and heat during blanching enhanced the starch gelatinisation that involved water uptake and starch swelling.

Figure 2 does not indicate the drying constant rate stage in the drying rate curve. This finding aligns with the study on celeriac by Wei et al. [24]. The entire drying process occurred in the falling rate stage, which implied that diffusion was the most likely a physical mechanism that regulates the moisture movement in celeriac slices [66].

The best-fitted model for celeriac slices drying for a total of 30 experiment runs was chosen to have the lowest SSE value among all mathematical models applied. Investigators use drying models to predict moisture content and drying time at given drying conditions, improve drying performance, and generalise drying kinetics for the design and operation of dryers [69]. Table 2 shows that the model by Midilli and others [70] and the logarithmic model [71] fitted best with celeriac slices drying kinetics. Doymaz et al. [66] also found both models as the most suitable mathematical models to describe the characteristics of tomato drying kinetics. Both models are derived from Fick’s second law of diffusion [64,72].

**Figure 3:**
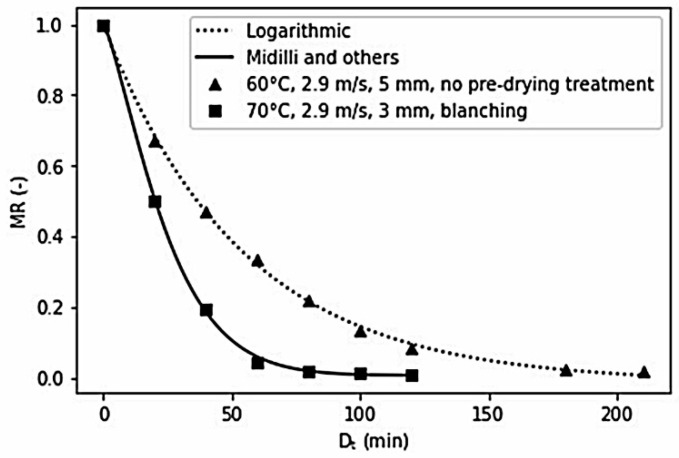
Drying kinetics of selected experiment runs and the corresponding best-fitted model.

Figure 3 shows the drying kinetics of selected experiment runs. The graph presents the changes in moisture ratio during drying and the corresponding mathematical model that fits the drying kinetics. In general, celeriac drying characteristics were like most agricultural products, and the pre-drying treatments did not seem to impact its drying behaviour.

### 3.2. Effect of Drying Factors on Hot Air-Dried Celeriac Slices Quality

This section presents the quality of dried celeriac slices, which includes physical and functional properties. Physical properties observed were chromaticity (*WI*, *BI*, ΔE*_ab_) and *RR*. Meanwhile, the functional properties studied were retentions of antioxidant activity (%*_AA_*) and total phenolic compound (%*_TPC_*).

#### 3.2.1. Chromaticity and Rehydration Ratio (*RR*)

Colour affects consumers’ preferences in choosing food products. It is interlinked with and may represent other properties of food. In addition, colour is controlled by reactions that occur during the supply chain. For these reasons, many researchers or food industries use this quality attribute to measure product quality [46]. Colour change in heat-treated foods may indicate the extent of corresponding quality degradation and estimate the heat exposure severity [73,74].

This study found the increase of *BI* and the decrease of *WI* of dried celeriac slices. *WI* indicates the intensity of whiteness in dried products [46], while browning is a critical phenomenon in food drying. The celeriac browning probably resulted from both enzymatic and non-enzymatic oxidation of phenolic compounds [46]. Analysis of total colour changes response help to optimise the food quality and process conditions [46]. Total colour difference (ΔE*_ab_) represents the magnitude of colour difference between fresh and dried samples. ΔE*_ab_ value of dried celeriac (ΔE*_ab_ > 3) in all experiment runs indicated the colour difference of dried samples was very distinct compared to the fresh samples [75].

**Figure 4:**
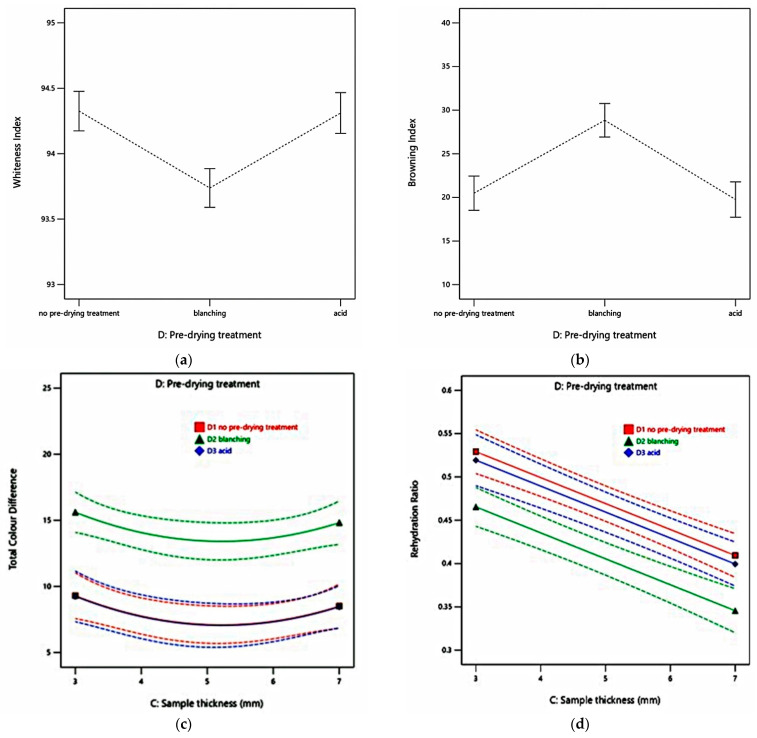
Physical properties as a function of factors: (**a**) *WI* of celeriac slices at different pre-drying treatments; (**b**) *BI* of celeriac slices at different pre-drying treatments; (**c**) ΔE*_ab_ of celeriac slices at different pre-drying treatments and sample thicknesses; (**d**) *RR* of celeriac slices at different sample thicknesses and pre-drying treatments.

This research utilised the food colour modelling approach to optimise the drying factors. Table 3 shows the equation of all chromaticity indices. Pre-drying treatment (*p* < 0.0001) affected all chromaticity indices (*WI*, *BI*, and ΔE*_ab_). Figure 4a–c depicts selected drying conditions that describe that blanched celeriac samples have significantly different colour properties than samples dipped in acid and samples without treatment before drying. Blanched samples have a higher ΔE*_ab_ and *BI*, and vice versa lower *WI* than samples with other pre-drying treatments. This finding aligns with the report on cocoyam samples, where pre-drying blanched samples had reduced *WI* and elevated *BI* compared to dried samples without blanching [68]. Furthermore, the removal of intercellular air affected the samples’ reflecting properties due to hot water blanching, resulting in translucent vegetables, and resulting in this noticeable shift of blanched samples’ colour characteristics [76]. Ben Zid et al. [77] mentioned the same prediction on blanched albedo (white part) of citrus peel where it became transparent and darker due to blanching water absorption and a decrease of *WI* was observed.

This research also considered the rehydration ratio (*RR*) an essential indicator in determining the quality of dried celeriac. The rehydration ratio represents the extent to which dried material undergoes physical and chemical damages [78]. The more severe the damage occurs on water binding compounds in the internal structure of materials, the lower the ability of dried material to reconstitute water, which is undesirable [79].

Figure 4d demonstrates that sample thickness and pre-drying treatments affected *RR* significantly (*p* < 0.0001). The *RR* decreased with the increase of sample thickness and blanching. The thinly sliced samples underwent a shorter exposure time during drying than the thicker samples because of a larger surface and volume ratio [69], thus possibly less damaged than thicker ones. In addition, the thin samples rehydrated more water than thicker samples; probably, water on the surface concentrated almost instantaneously, led by rapid rehydration of capillaries and cavities close to the surface [80]. Another study on hot air-dried cocoyam indicated samples with four mm thickness feature the highest value of *RR* and decreased to the lowest value at 10 mm thickness [68]. Furthermore, this study found that blanched samples had a lower *RR* value, probably due to damage that occurred during drying in the form of irreversible cellular rupture and disintegration. These resulted in dense, considerably shrunken collapsed capillaries that had reduced hydrophilic capacity, reducing the ability of biological material to imbibe water [81].

**Figure 5:**
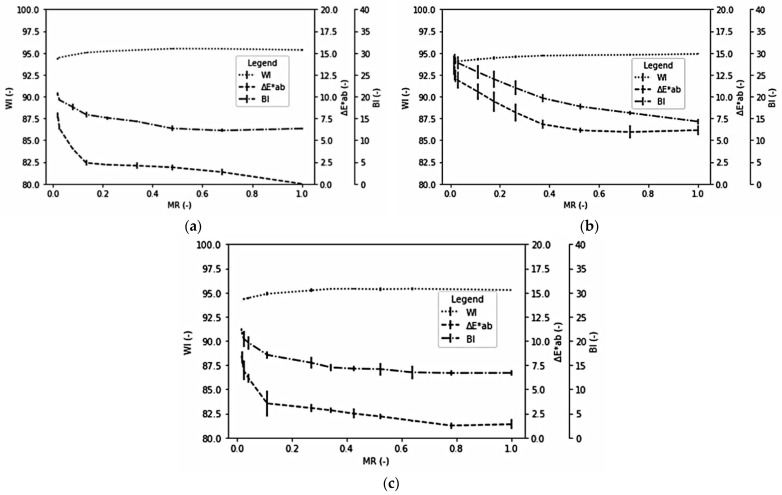
Dynamic changes of chromaticity indices as a function of MR at 60 °C drying temperature at (**a**) 2.9 m/s air velocity, 5 mm sample thickness, no pre-drying treatment; (**b**) 2.2 m/s air velocity, 5 mm sample thickness, blanching pre-drying treatment; (**c**) 2.2 m/s air velocity, 7 mm sample thickness, acid pre-drying treatment.

Moisture content affected the reflectance colour values of dried samples [82]. Figure 5 shows the dynamic changes of chromaticity as a function of *MR* for each selected pre-drying treatment. The blanched celeriac slices perform higher ΔE*_ab_ and *BI* than the other two pre-drying treatments throughout the drying process. Shrestha et al. [83] also found that blanching was ineffective in preventing browning in apple slices under study. The authors argued that the blanching endorsed cellular integrity loss degraded natural pigments, which, in turn, enhanced the browning rate.

Blanched celeriac slices had a relatively stable ΔE*_ab_ and the *BI* in the initial phase of the drying process. Then, however, the value started to rise at *MR* below 0.4. The other two treatments initially increased slowly and began to increase sharply at *MR* below 0.1. Ndisya et al. [68] also had similar findings in drying blanched cocoyam. Therefore, they concluded that the blanching stage denatured heat-labile enzymes. At the same time, the heat-stable part dominated the colour changes during the drying process, together with optical properties changes, because of starch gelatinisation, resulting in the consistent reduction in lightness (increase in ΔE*_ab_, and the *BI*, lower *WI*).

#### 3.2.2. Antioxidant Activity (AA) and Total Phenolic Compound (TPC)

**Figure 6:**
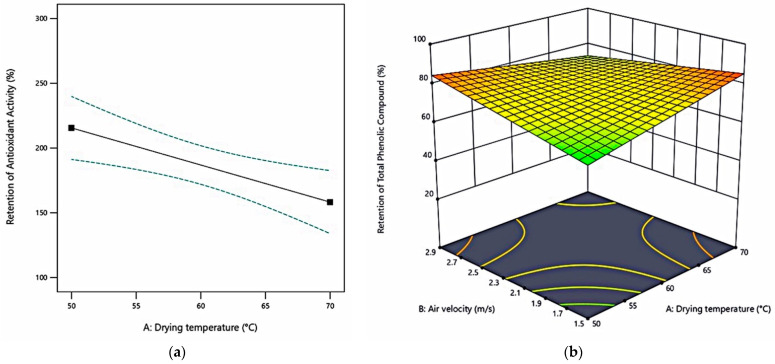
Functional properties function of factors: (**a**) %*_AA_* at different drying temperatures; (**b**) %*_TPC_* at different drying temperatures and air velocities, 3 mm sample thickness, no pre-drying treatment.

The average initial antioxidant activity of fresh celeriac slices was 5.63 ± 1.32 µmol TE/g_ds_ and increased to 7.95–10.94 µmol TE/g_ds_ after drying. The average initial total phenolic compound of fresh celeriac slices was 1.16 ± 0.17 mg GAE/g_ds_. The TPC value decreased to 0.37–1.12 mg GAE/g_ds_ after drying. Figure 6 presents the retention of antioxidant activity (%*_AA_*) and retention of total phenolic compound (%*_TPC_*).

This study found that dried samples compared with fresh samples increased AA values but decreased TPC values. This finding is in agreement with Madrau et al. [84], who studied apricot. They explained that the intermediate oxidation state of polyphenols, the increasing number of reducing sugar, and the Maillard reaction products formation resulted in a decrease in polyphenols and increased antioxidant activity. Heat treatment may trigger non-enzymatic browning that produces partially oxidised polyphenol with higher antioxidant activity capacity than non-oxidised phenols [85]. Lourenço et al. [86] also observed no correlation between TPC and AA in the spray-dried natural antioxidant extracts from pineapple peel. They hypothesised that it is probably due to the varied effectiveness among different phenolic compounds as antioxidants [87]. Figure 6a,b describe the model of %*_AA_* and %*_TPC_* of not pre-drying treated celeriac slices, respectively. The figures showed decreased %*_AA_* when higher drying temperature was applied; however, the %*_TPC_* of not pre-drying treated samples was relatively stable over the experimental region. Setyawan et al. [88] also found that oven-dried samples without blanching or water-steeping before drying had the highest total phenolic compounds of yam flour because such pre-drying treatments caused the loss of phenolic compounds, which were water-soluble.

### 3.3. Effect of Drying Factors on Hot Air-Dried Celeriac Slices Energy Consumption (E_s_)

**Figure 7:**
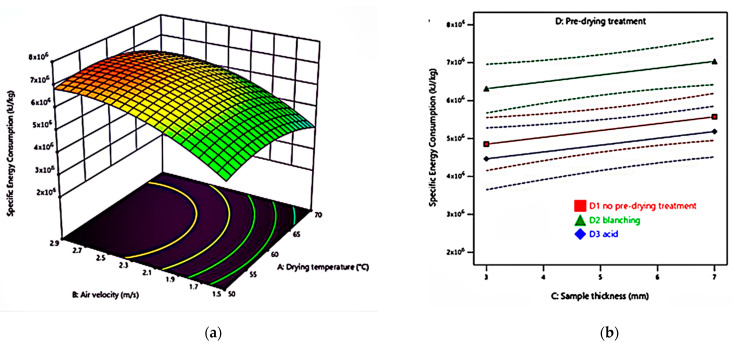
(**a**) Specific energy consumption of 5 mm sample thickness, blanching pre-drying treatment celeriac slices hot air at different drying temperatures and air velocities; (**b**) specific energy consumption of different sample thicknesses and pre-drying treatments of celeriac slices hot air at 60 °C of drying temperature and 2.2 m/s of air velocity.

The lowest specific energy consumption of 2.7 × 10^6^ kJ/kg (*D_t_* = 180 min) belonged to an experiment run at 50 °C, 1.5 m/s, 3 mm sample thickness without pre-drying treatment. Meanwhile, the highest specific energy consumption of 7.7 × 10^6^ kJ/kg belonged to the experiment run at 60 °C, 2.2 m/s, 5 mm sample thickness, and blanching pre-drying treatment (*D_t_* = 420 min). Figure 7 shows the specific energy consumption as a function of drying factors.

To evaluate drying performance, investigators must consider drying efficiency that involves drying time, drying rate, and energy consumption simultaneously [30]. Table 3 and Figure 7a show *E_s_* initially increased with an increment in drying temperature up to around 60 °C and declined afterwards because of the quadratic effect of this factor. This result agrees with a cocoyam study because an increase in the drying rate and a reduction in the drying time compensate for the increment in drying temperature [68]. An increase in air velocity increased energy consumption, but the quadratic air velocity affected lowering energy consumption. This result is in line with the thin-layer drying of the Jujube study report, as increasing the air velocity decreases the drying time [89].

Figure 7b shows an increase in sample thickness directly proportional to the *E_s_* magnitude, and this was also the case with the blanched samples. This phenomenon occurred because a relatively smaller surface area to volume ratio that held more moisture while blanching led to the hard surface creation resulting from starch gelatinisation that prevented the water removal [68]. Both factors, the thicker or blanched samples, required longer drying time and, thus, had increased *E_s_*.

### 3.4. Optimisation

The experiment design is relevant to experimentation at various scales, from laboratory to industry, and quality improvement [90]. This study demonstrated optimisation by considering important aspects for manufacturers (drying time and specific energy consumption) and consumers (functional and colour properties). This study applied the maximum importance level (scale 5) for all the responses of interest for both stakeholders in the desirability function. However, this method only considered post-process response without involving the products’ dynamic changes during the drying process.

**Figure 8:**
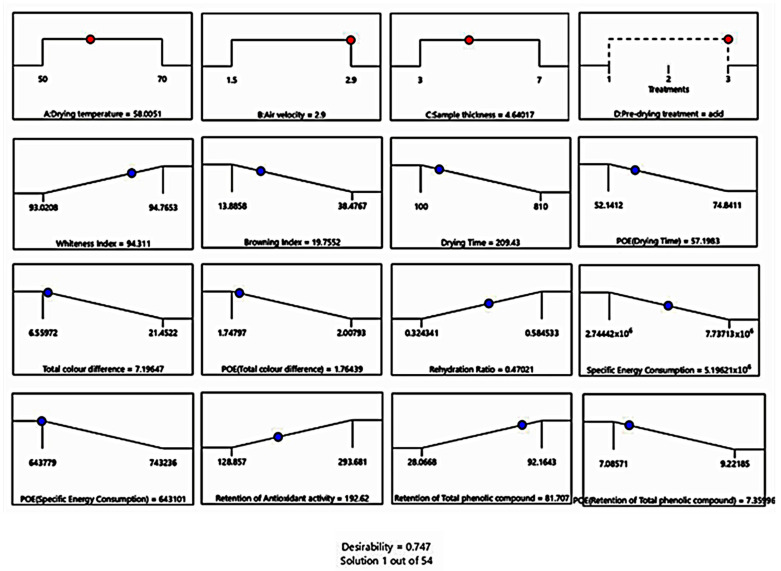
Numerical optimisation for quality and specific energy consumption of hot-dried celeriac slices.

Optimisation of the processing period and pre-drying treatment are essential to produce high-quality products [83]. Figure 8 outlines the selected solution with the corresponding predicted response level. The drying temperature of 58 °C, air velocity of 2.9 m/s, sample thickness of 4.6 mm with acid pre-drying treatment gave a fair optimum desirability result for *E_s_*, *RR*, and %*_AA_* but high optimum desirability for *D_t_*, chromaticity, and %*_TPC_*. The selected factor setting did not necessarily satisfy the maximum value of all responses but provided a balance between conflicting responses [68]. Today, the development of post-harvest quality inspection approaches is, in particular, targeted on their optimisation, making use of new opportunity techniques, which lessen processing times, decrease waste, and attain extra standardisation in their products [91]. One prospective technique is an in-process non-invasive quality inspection during drying that will also be considered for further studies.

Table 3 shows the coefficients of selected models with coded factors. As the model contains a categoric factor (pre-drying treatment, x_4_), there are two terms of x_4_ (x_4_ (1) and x_4_ (2)), which is one less term than the number of levels in the factor. The product of the number of terms in the main effects produces the number of interaction terms. To predict the first level of x_4_ (no pre-drying treatment), substitute +1 for x_4_ (1) and 0 for x_4_ (2). For the second level (blanching), substitute 0 for x_4_ (1) and +1 for x_4_ (2). For the third level (acid dipping), substitute −1 for x_4_ (1) and −1 for x_4_ (2)] [92].

## 4. Conclusions

The experiment used the I-optimal design of response surface methodology to investigate the optimisation of hot air-drying celeriac slices’ quality and energy consumption. As predicted, *D_t_* was directly proportional to the thickness of the sample (*p* < 0.0001), but, with the increase of the temperature (*p* < 0.0001) and drying air velocity (*p* < 0.001), it declined. Blanching increased the drying time because it enhanced water uptake and starch swelling, increasing the initial moisture content. Pre-drying treatment (*p* < 0.0001) affected all chromaticity indices (*WI*, *BI*, and ΔE*_ab_). Blanched samples had a higher ΔE*_ab_ and *BI* and lowered *WI* than samples with other pre-drying treatments. Sample thickness and pre-drying treatments affected *RR* significantly (*p* < 0.0001). In general, celeriac drying characteristics are like most agricultural products, and pre-drying treatments did not impact its drying behaviour. The model by Midilli and others and the logarithmic model fitted best with celeriac slices drying kinetics. The *RR* decreased with the increase of sample thickness and blanching. The thin samples rehydrated more water than thicker samples, probably because of the rehydration of capillaries and cavities close to the surface. In addition, the thin samples underwent minor damage because of shorter drying exposure. In addition, blanching caused cellular damage that prevented biological material from imbibing water. This study also found that dried slices compared with fresh slices increased AA value but decreased TPC value. Heat treatment may trigger non-enzymatic browning that produced partially oxidised polyphenol with higher antioxidant activity capacity than non-oxidised phenols.

*E_s_* initially increased with an increment in drying temperature up to around 60 °C and declined afterwards because of this factor’s quadratic effect. An increase in air velocity increased *E_s_*, but the quadratic air velocity affected lowering energy consumption. A smaller surface area to volume ratio in thicker slices that held more moisture while blanching led to the hard surface creation resulting from starch gelatinisation that prevented the water removal. Both factors, the thicker or blanched samples, required longer drying time and, thus, have increased *E_s_*. The solution chosen for optimal responses was the drying temperature of 58 °C, air velocity of 2.9 m/s, and a sample thickness of 4.6 mm with acid pre-drying treatment. This study considered parameters that were critical for manufacturers and consumers for optimisation. The optimum drying factors were a balance between competing responses. Optimisation utilising the experiment design is relevant in quality improvement and is applicable in the food industry scale. However, this method only considered post-process response without involving the products’ dynamic changes during the drying process. One prospective post-harvest quality inspection technique is an in-process non-invasive quality inspection during drying that will also be considered for further studies.

## Figures and Tables

**Table 1:** Factors and actual level corresponding with the coded level.

Factors	Coded Factors	Level		
-	-	−1	0	+1
Drying temperature (°C)	x_1_	50	60	70
Air velocity (m/s)	x_2_	1.5	2.2	2.9
Sample thickness (mm)	x_3_	3	5	7
Pre-drying treatment	x_4_	N	B	A

N is without pre-drying treatment, B is blanching, A is acid dipping.

**Table 2:** The mathematical models fitted best the celeriac slices drying kinetics.

Model	Frequency	Model Formula	Reference
Midilli and others	24	$MR=a\times e^{\left(-k\left(t^{n}\right)\right)}+b\times t$	[70]
Logarithmic	6	$MR=a\times e^{\left(-kt\right)}+c$	[71]

**Table 3:** Quality parameters and specific energy consumption of celeriac slices subjected to different drying temperatures, air velocities, sample thicknesses, and pre-drying treatments.

-	*D_t_* (min)	*WI*(-)	*BI*(-)	ΔE*_ab_(-)	*RR*(-)	*E_s_* (kJ/kg)	%*_AA_* (%)	%*_TPC_* (%)
Intercept	281.570	94.125	23.025	9.093	0.4447	5,590,060.360	186.909	66.633
x_1_	−101.174 ^1^	-	-	-	-	−310,381.836 ^2^	−28.629 ^2^	2.760 ^2^
x_2_	−34.327 ^2^	-	-	-	-	1,076,236.090 ^1^	-	−3.152 ^3^
x_3_	127.946 ^1^	-	-	−0.395 ^4^	−0.0600 ^1^	359,674.738 ^2^	-	−2.548 ^4^
x_4_ (1)	−33.874 ^1^	0.201 ^2^	−2.544 ^1^	−2.086 ^1^	0.0245 ^1^	−358,525.812 ^1^	-	−2.496 ^2^
x_4_ (2)	73.962 ^1^	−0.387 ^2^	5.814 ^1^	4.224 ^1^	−0.0392 ^1^	1,104,370.500 ^1^	-	−4.054 ^2^
x_1_ x_2_	-	-	-	-	-	-	-	−7.348 ^2^
x_1_ x_3_	−44.081 ^2^	-	-	-	-	-	-	8.812 ^2^
x_1_ x_4_ (1)	-	-	-	-	-	-	-	7.972 ^2^
x_1_ x_4_ (2)	-	-	-	-	-	-	-	2.118 ^2^
x_2_ x_3_	−37.198 ^2^	-	-	-	-	-	-	-
x_2_ x_4_ (1)	-	-	-	-	-	-	-	4.624 ^2^
x_2_ x_4_ (2)	-	-	-	-	-	-	-	−12.379 ^2^
x_3_ x_4_ (1)	-	-	-	-	-	-	-	−10.827 ^2^
x_3_ x_4_ (2)	-	-	-	-	-	-	-	12.038 ^2^
x_1_ ^²^	-	-	-	-	-	−539,553.369 ^2^	-	-
x_2_ ^²^	-	-	-	-	-	−680,688.662 ^2^	-	-
x_3_ ^²^	-	-	-	1.788 ^2^	-	-	-	-
Model F value	31.189 ^1^	11.454 ^2^	15.160 ^1^	26.740 ^1^	36.810 ^1^	17.742 ^1^	9.339 ^2^	8.833 ^1^
Lack of Fit F value	3.035 ^4^	1.368 ^4^	1.836 ^4^	1.650 ^4^	2.210 ^4^	1.722^4^	2.276 ^4^	0.497 ^4^
R ^2^ adj	0.879	0.419	0.494	0.780	0.787	0.802	0.223	0.778

^1^*p* < 0.0001, ^2^ *p* < 0.05, ^3^ 0.05 ≤ *p* ≤ 0.1, ^4^ *p* > 0.1, - not significant model terms (not counting those required to support hierarchy); x_1_ = drying temperature (°C), x_2_ = air velocity (m/s), x_3_ = sample thickness (mm), x_4_ = pre-drying treatment; x_4_ (1) and x_4_ (2) are terms of the x_4_ (categoric) factor.

## Data Availability

Not applicable.
